# Kidney Injury under Diminished Pulsatile Flow Induced by V-A ECMO in Sheep

**DOI:** 10.7150/ijms.103349

**Published:** 2025-01-27

**Authors:** Weidong Yan, Tianlong Wang, Jiachen Qi, Luyu Bian, Shengqiang Pei, Qiaoni Zhang, Sizhe Gao, Shujie Yan, Gang Liu, Yuan Teng, Jian Wang, Chun Zhou, Qian Wang, Jing Wang, Bingyang Ji

**Affiliations:** 1 Department of Cardiopulmonary Bypass, Fuwai Hospital, National Center for Cardiovascular Disease, State Key Laboratory of Cardiovascular Medicine, Chinese Academy of Medical Science & Peking Union Medical College, 100037 Beijing, China.; 2 Department of Anesthesiology, Beijing Chao-Yang Hospital, Capital Medical University, Beijing 100022, China.; 3 Department of Pain Medicine, Beijing Tsinghua Changgung Hospital, School of Clinical Medicine, Tsinghua University, 102218 Beijing, China.; 4 State Key Laboratory of Cardiovascular Disease, Beijing Key Laboratory for Molecular Diagnostics of Cardiovascular Diseases, Diagnostic Laboratory Service, Fuwai Hospital, National Center for Cardiovascular Diseases, Chinese Academy of Medical Sciences & Peking Union Medical College, 100037 Beijing, China.

**Keywords:** Extracorporeal membrane oxygenation, Diminished pulsatile flow, Kidney injury

## Abstract

Extracorporeal membrane oxygenation (ECMO) is commonly used for critically ill patients and associated with high rates of kidney injury and mortality. This study aimed to assess the impact of diminished pulsatile flow induced by veno-arterial ECMO (V-A ECMO) on kidney structure and function. Ten healthy sheep were divided into two groups (n=5 each) to compare diminished pulsatile flow induced by V-A ECMO with normal pulsatile flow under veno-venous ECMO (V-V ECMO) over 7 days. Vital signs were recorded every 6 hours, and renal biomarkers were tested every 24 hours. The pulse pressure (PP) in the V-A ECMO group was around 20 mmHg, compared to 30 mmHg in the V-V ECMO group. The pulsatility index (PI) was lower than 0.25 in the V-A ECMO group and higher in the V-V ECMO group. Mixed model results showed significant differences in PP and PI between the two groups (p = 0.044 and p = 0.016, respectively). BUN and Cr levels were similar in both groups, but Cystatin C was higher in the diminished pulsatile flow group. The expression of anti-platelet endothelial cell adhesion molecule-1 (CD31) and the renin-positive glomerulus ratio were significantly lower in the diminished pulsatile flow group. Both groups exhibited hypoxia-inducible factor-1α (HIF-1α) in renal tubules, indicating hypoxia. These findings suggest diminished pulsatile flow under V-A ECMO affects renal endothelial cell proteins and renin secretion. During the management of ECMO, although the serum BUN and Cr were normal, there was exactly an early-stage injury of the glomerulus and tubules in both groups.

## Introduction

Extracorporeal membrane oxygenation (ECMO) is widely used for critically ill patients and associated with a high incidence of kidney injury and mortality^1^, ^2^. The pathophysiology of kidney injury during ECMO is multifactorial, including reperfusion after ischemia, alteration of renal microvasculature, and oxidative stress^3^-^6^. Besides, the pulsatility of blood flow is one of the factors influencing microcirculation^7^-^9^. Several studies have demonstrated that pulsatile flow is more beneficial than non-pulsatile flow and it has great impact on the circulatory system, endothelial cells, and end-organ perfusion^10^, ^11^. It should be noticed that the blood flow under veno-arterial ECMO (V-A ECMO) support is continuous flow or diminished pulsatile flow, which depends strongly on the amount of its cardiac output versus ECMO flow.

The controversial debate over the benefits of pulsatile and non-pulsatile flow has been raised with ECMO development^12^-^14^. During ECMO, maintaining pulsatile flow can provide certain benefits to patients, but it requires invasive procedures or specialized pulsatile pumps. Clinically, it is more common for patients to have a diminished pulsatile flow during ECMO support, as their heart function is not completely lost. So, the purpose of this study was to figure out whether the diminished pulsatile flow induced by V-A ECMO would influence both the structure and function of the kidney. Among the existing ECMO-related experiments, the effect of a single variable could not be strictly achieved which would lead to some bias. At the same time, the long-term impact of diminished pulsatile flow could not be revealed with the fact that most of the observation time was less than 48 hours^12^, ^15^. Hence, in this study, healthy sheep with normal cardiac function were selected to establish two ECMO modes. After guaranteeing the same experimental conditions for 7 days, the effects of the V-A ECMO diminished pulsatile flow on the kidney were compared with normal pulsatile flow induced by veno-venous ECMO (V-V ECMO).

## Methods

### Animal and groups

The Institutional Animal Care and Use Committee of Fuwai Hospital approved this study (0101-2-20-HX-X). This experiment was conducted at Beijing Key Laboratory of Pre-clinical Research and Evaluation for Cardiovascular Implant Materials, Animal Experimental Center of Fuwai Hospital. All the procedures were in accordance with the Guide for the Care and Use of Laboratory Animals published by the National Institutes of Health, USA. The whole study followed the ARRIVE guidelines (http://www.nc3rs.org.uk/arrive).

Adult (12~14-month-old) healthy male Small Tailed Han sheep weighing between 54 kg and 63 kg were utilized in this study (Purchasing from Beijing Jinyutongfeng Trading Co., Ltd., Beijing, China). Use random numbers to group the sheep. All sheep suffered the implantation of the ECMO system (Jiangsu STMed Technologies Co., Suzhou, China), which was described in detail in the previously published article^16^. Briefly, the ECMO system consisted of a control console, a pump drive, and a centrifugal pump. Besides, the membrane oxygenator with hollow polymethyl pentene fibers (Hilite7000LT, XENIOS, Heilbronn, Germany; BE-PLS 2050, Maquet, Rastatt, Germany) was randomly used. Sheep were randomly divided into two groups. Sheep cannulated with the common carotid artery and external jugular vein were assigned to the V-A ECMO group. The others cannulated with internal jugular vein using a double-lumen cannula (DLC) were assigned to the V-V ECMO group. To guarantee comparable conditions between the experimental groups, exclusion criteria were defined. Sheep were excluded from the study when ≥1 of the following conditions were fulfilled during the experimental procedure: low hemoglobin concentration <5.0 g/L, persistent low mean arterial pressure <50 mmHg not responding to norepinephrine application, low partial pressure of arterial oxygenation <80 mmHg.

### Prepare for canulation

The details of the protocol were previously published^17^. After 48 hours of fasting and 12 hours of water deprivation, sheep were injected with propofol (3 - 5mg/kg) for the induction of anesthesia and intubated with an endotracheal tube. Then mechanical ventilation was conducted with the tidal volume of 8 - 10 ml/kg, and the respiratory rate was 12 - 20 breaths/min. General anesthesia was maintained with isoflurane (2% - 3%) and propofol (8 - 10 mg/kg/h). The left common carotid artery was used for hemodynamic monitoring and blood gas analysis. The left jugular vein was used for drug injection and blood collection. After the vital signs were stable, the sheep were immobilized on the operating table in a supine position. Surgeons exposed the artery and vein carefully. Systemic anticoagulation was induced with heparin (120 UI/kg). When the target activated clotting time (ACT) was higher than 250 s, cannulation was performed.

### Cannulation and initiation of ECMO

The details of the establishment and management of the ECMO sheep model have been published^18^. The key points are described below.

In the V-A ECMO group, an 18-Fr arterial cannula (Edwards Lifescience, Irvine, CA, USA) was inserted into the right common carotid artery with the cannula descending 10 - 15 cm and a 24-Fr venous cannula (Edwards Lifescience, Irvine, CA, USA) was inserted into the right atrium through the right external jugular vein. In the V-V ECMO group, a 23-Fr DLC (Maquet, Rastatt, Germany) was inserted into the right internal jugular vein with the tip positioned in the inferior vena cava confirmed by transthoracic echocardiography. The selection of the size of all cannulas was collaboratively determined by veterinarians, clinicians, and ECMO equipment engineers. Then the cannulas were connected to the pump and oxygenator which was primed with priming solution (700 mL). The components of the priming solution were lactated Ringer's solution. Air was carefully removed from the system then extracorporeal circulation was initiated with a pump flow of 2.0 - 2.5 L/min and a rotational speed of 3,200 - 3,500 rpm.

### ECMO management

The sheep were moved into a cage and properly restrained after the vital signs were stable. The concentration of isoflurane was decreased gradually and the sheep was extubated after 30 minutes of spontaneous respiration and documentation of satisfactory blood gases and acid-base balance. In the first 24 hours, dexmedetomidine (0.2 - 0.3 μg/kg/h) and flurbiprofen (1 - 2 mg/kg) was administered intravenously. Then the sheep stayed awake in the cage and freely ate and drank. Heparin was infused continuously to maintain the ACT in the range of 220 -250s. The pump flow and speed were monitored throughout the whole experiment and adjusted according to the blood gas analysis results and vital signs. The optimal flow during the experimental period for each sheep was determined by the mean arterial pressure (MAP) at baseline. If the mean arterial pressure (MAP) is below 50 mmHg, a temporary dose of norepinephrine (8 µg per dose) will be administered. The vasoactive drug was not continuously used in the management of MAP during the whole procedure. If the sheep needed vasoactive drugs to maintain the MAP, they were excluded. The sheep were supported on ECMO for 168 hours, then the sheep were euthanized by administration with potassium chloride (100 mg/kg) under deep anesthesia (propofol 20 mg/kg). There was no difference in the management of ECMO in both groups.

### Parameters of diminished pulsatile flow

Under both ECMO support modes, the pulsatility was synchronized with the heart rate. In the V-A ECMO group, oxygenated blood was infused into the aortic arch via the right common carotid artery, which was equivalent to unloading a portion of the cardiac blood flow and reducing the original cardiac stroke volume, thereby leading to a decrease in blood pulsatility. In contrast, in the V-V ECMO group, oxygenated blood was directly infused into the right atrium, which did not affect the normal cardiac ejection, and thus had little impact on its pulsatility. The pulse pressure (PP) and pulsatility index (PI) were calculated based on the blood pressure, which were used to identify the degree of blood flow pulsatility. The PP equals to the systolic blood pressure minus the diastolic blood pressure. The PI is equal the difference in pulse pressure divided by the mean arterial pressure. There is a study that defined low pulse flow as PP < 20mmHg^19^, while in our study, we defined diminished pulsatile flow as PP around 20mmHg.

### Primary outcome

This study aimed to evaluate the effect of diminished pulsatile flow on the kidney compared with normal pulsatile flow induced by V-V ECMO. The structure and function of the kidney were assessed. Endothelial cell markers CD31 and renin were investigated to evaluate the renal epithelial cell structure and renal tissue function. Blood urea nitrogen (BUN), creatinine (Cr), and cystatin C (CyC) were tested as markers of glomerular filtration function. The NGAL and HIF-1α were assessed for evaluating the kidney injury.

### Data collection

We collected baseline characteristics, including weight, the concentration of hemoglobin (Hb), BUN, and Cr. Blood pressure and heart rate (HR) from the bedside monitors were recorded every 6 hours during the 168 hours following ECMO cannulation. Additionally, ECMO characteristics, such as pump speed, flow rate, pre-pump pressure, post-pump pressure, and ACT were monitored serially. The following laboratory values were obtained as the baseline values at the initiation of ECMO and every 24 hours during the experiment: BUN, Cr, and serum K^+^, Na^+^, Cl^-^, and Ca^2+^.

### Histology

After weaning from ECMO, the renal tissue samples were stained with hematoxylin-eosin to evaluate the structure of the glomerular and tubules. All the tissue samples had been fixed in 4% buffered formalin immediately after euthanasia for 2 days and then embedded in paraffin. Slides of 4 μm thickness were stained with hematoxylin and eosin according to standard procedures. Sections were permeabilized with 0.1% Triton X-100(ZSGB-Bio, China) and incubated with 10% goat serum (ZSGB-Bio, China). Immunohistochemistry was used for the detection of anti-platelet endothelial cell adhesion molecule-1 (PECAM-1/CD31), renin, and neutrophil gelatinase-associated lipocalin (NGAL) protein. Sections were incubated with rabbit anti-CD31 antibody (ZA-0568, ZSGB-Bio, China), rabbit anti-renin antibody (ab212197, Abcam, UK), and rabbit anti-NGAL antibody (ab216462, Abcam, UK) overnight at 4 ℃. Then, they were incubated with the enzyme-conjugated goat anti-rabbit IgG polymer in a rabbit two-step kit (ZSGB-Bio, China). Immunofluorescence was used for the detection of hypoxia-inducible factor-1α (HIF-1α). Sections were further incubated with anti-HIF-1α antibody (36169, Cell Signaling Technology, USA) overnight at 4 ℃ followed by additional incubation with Cy3-conjugated goat anti-rabbit IgG (1:300, Servicebio, China) for 1 h at room temperature. Nuclei were counterstained with 4',6-diamidino-2-phenyl indole (DAPI, ZSGB-Bio, China). Sections were screened using Pannoramic SCAN (3D HISTECH, Hungary) and analyzed using ImageJ software (National Institutes of Health, Bethesda, USA).

### Statistics

Numerical data are presented as the mean ± standard deviations (SD). Student's t-test was used to investigate the differences between the two groups. The Welch's correction or the Mann-Whitney U-test was used if the data did not pass the F-test or the normality test. The linear mixed model was used to compare the pulse pressure and pulsatility index, and it was conducted with R (v.4.2.2) by using the “nlme” package. A *p* value < 0.05 (2- tailed) was considered statistically significant.

## Results

### The management of the two groups

Finally, ten sheep were enrolled after excluding two sheep in the V-A ECMO group, and there were 5 sheep in each group. The two excluded sheep died within 48 hours after ECMO implantation. One suffered persistent low partial pressure of arterial oxygen (PaO_2_, 62 mmHg) and died of respiratory and circulatory failure. The other one suffered extremely decreased hemoglobin and died of hemorrhagic shock. The autopsy confirmed that fungal pneumonia and bleeding at the left common artery cannulation site were the cause of death, respectively. All of the other sheep were observed for 168 hours and autopsies were performed.

All baseline characteristics of sheep in the two groups are shown in **Table 1**. The average HR before the initiation of ECMO in both groups of sheep was 114.00 ± 28.59 vs. 142.00 ± 12.39 beats per minute (V-A vs. V-V), while MAP was 93.00 ± 9.30 vs. 88.40 ± 9.50 mmHg (V-A vs. V-V). The concentration of Hb of the V-A ECMO group was lower than that of the V-V ECMO group (93.60 ± 17.73 vs. 126.20 ± 24.69 g/L). The baseline serum BUN and Cr were similar in both groups (V-A ECMO vs V-V ECMO; BUN, 6.27 ± 2.06 vs 5.39 ± 1.50 mmol/L; Cr, 158.57 ± 35.00 vs 134.94 ± 14.03 μmol/L). At 6 hours of ECMO, there was no difference among the parameters associated with ECMO (pump speed, flow, post-pump pressure). The pre-pump pressure of V-A ECMO was lower than V-V ECMO (-45.00 ± 5.94 vs. -66.00 ± 3.67). The ACT was stable in both groups during the whole procedure. When weaning from ECMO, both the parameters associated with ECMO and vital signs were similar in each group except for the pump speed, which was lower in the V-A ECMO group.

### The diminished pulsatile flow of the V-A ECMO group

**Figure 1:**
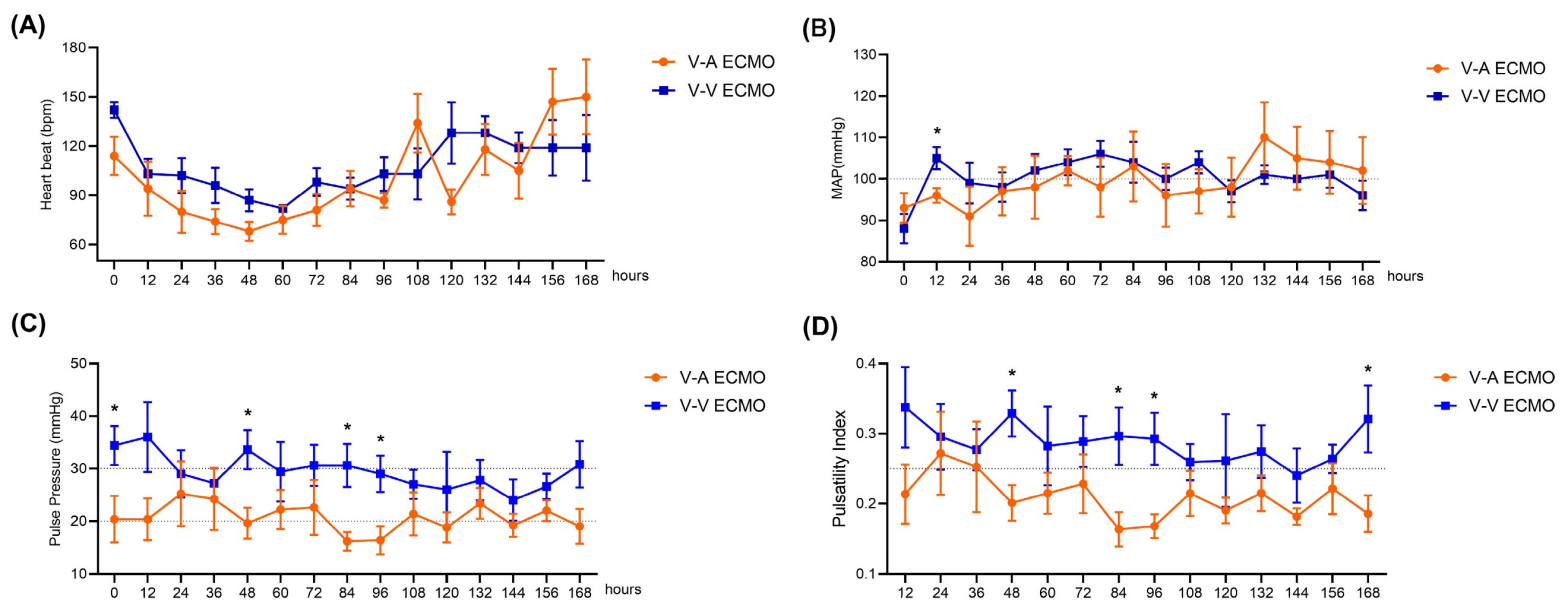
** Vital signs of the ECMO model.** Hear beat (A) and mean arterial blood pressure (B) were monitored throughout the experiment and recorded every 12 hours. Pulse pressure (C) equaled to the difference between systolic blood pressure and diastolic blood pressure. Pulsatility index (D) equaled to the ratio of pulse pressure to mean arterial pressure. V-A ECMO: veno-arterila extracorporeal membrane oxygenation; V-V ECMO: veno-venous extracorporeal membrane oxygenation; MAP: mean arterial pressure. * *p* < 0.05; *p* > 0.05 was not shown.

**Figure 2:**
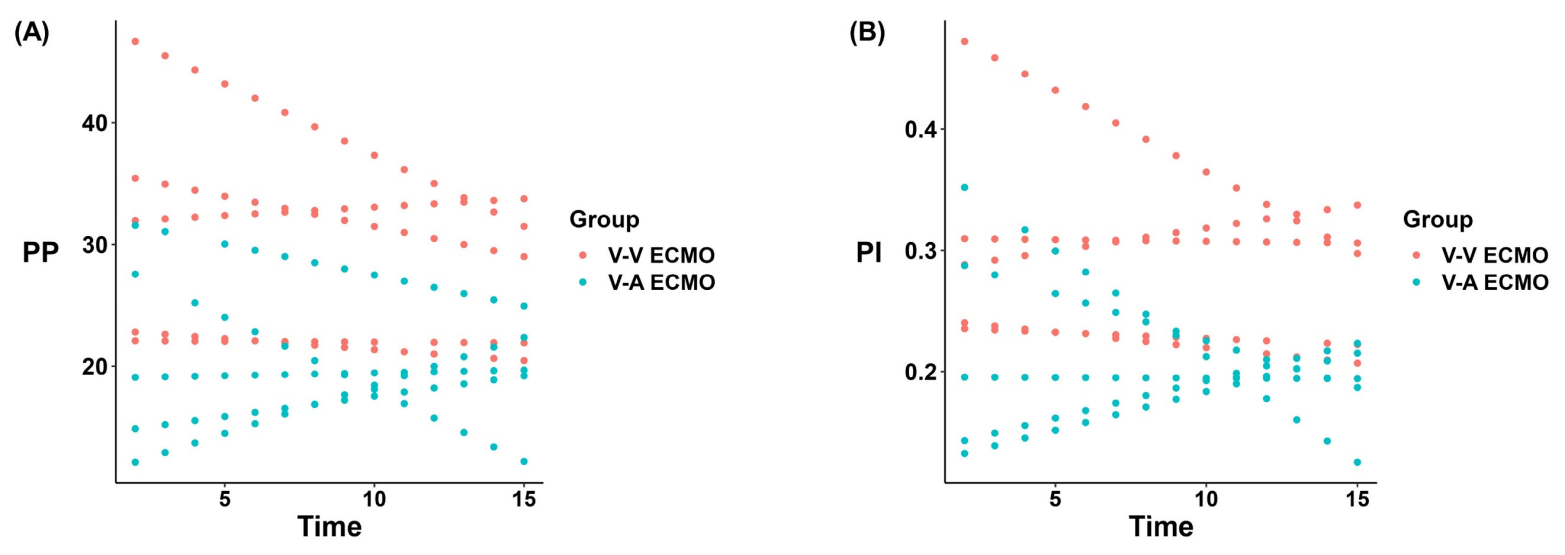
** Liner mixed model results of the pulse pressure and pulsatility index.** PP: pulse pressure, PI: pulsatility index, V-A ECMO: veno-arterila extracorporeal membrane oxygenation; V-V ECMO: veno-venous extracorporeal membrane oxygenation.

A representative recording of HR and MAP for each group is presented in **Figure 1**. During the 168-hour ECMO management, both groups showed the same trend in HR and MAP** (Figure 1A-B)**. Only at 12-hour ECMO run, the MAP of the V-V ECMO group was slightly higher than that in the V-A ECMO group (105 ± 6 vs. 96 ± 4 mmHg, *p* = 0.023). The MAP fluctuated around 100 mmHg in both groups throughout. Generally, the PP of the V-A ECMO group fluctuated around 20 mmHg, while it of the V-V ECMO group went ground 30 mmHg** (Figure 1C)**. The PI of V-A ECMO groups was lower than 0.25 and it was higher than 0.25 in V-V ECMO group **(Figure 1D)**. The statistical comparison results of the above basic vital signs are shown in **Table S1**. Considering that the measurement values of different individuals had different slopes over time, we applied a mixed linear model to compare the PP and PI. As shown in** Figure 2**, we fixed the slopes of the measurement values for each individual over time and conducted a comparison between groups. PP and PI were both significantly different in the two groups (p = 0.044 and p = 0.016, respectively)** (Table S2)**. Combined with PI, we defined that the blood flow in the V-A ECMO group was a diminished pulsatile flow.

### Diminished pulsatile flow decreased the CD31 and renin

**Figure 3:**
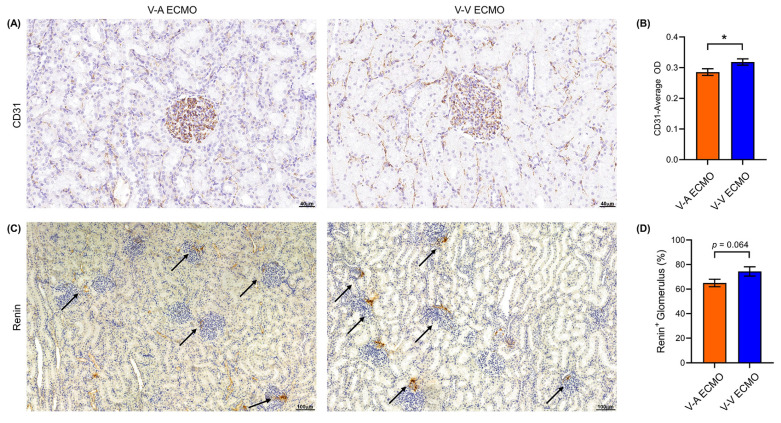
** The vascular endothelial cell marker (CD31) and renin expression in ECMO.** (A) The expression of vascular endothelial cell markers (CD31) was down-regulated in V-A ECMO. Three fields were randomly selected for each kidney specimen (n = 4, each group) and the average optical density (OD) was calculated using the Image J software. Scale bar: 40μm. The semi-quantitative expression of CD31 (B) in V-A ECMO group was lower than that of the V-V ECMO group. (C) The secretion of renin is reduced in V-A ECMO. Scale bar: 100μm. Three fields were randomly selected for each kidney specimen (n = 5, each group) and (D) the ratio of rennin positive glomerulus to the total number of glomerulus in the V-A ECMO group was calculated. The t-test was used for comparison between groups. V-A ECMO: veno-arterila extracorporeal membrane oxygenation; V-V ECMO: veno-venous extracorporeal membrane oxygenation. * *p* < 0.05; *p* > 0.05 was not shown.

To evaluate the pulsatility of blood flow on the renal epithelial cell structure and the function of renal tissue, the expression of endothelial cell marker CD31 and renin was evaluated by immunohistochemical staining of the kidney tissue. The expression of CD31 in the diminished pulsatile flow group was significantly lower than that in the normal pulsatile flow group** (Figure 3A-B)**. The ratio of the renin-positive glomerulus of the diminished pulsatile flow group was also lower than the normal group, but without statistical difference (p = 0.064) **(Figure 3C-D)**.

### The concentration of cystatin C was increased in the diminished pulsatile flow group

**Figure 4:**
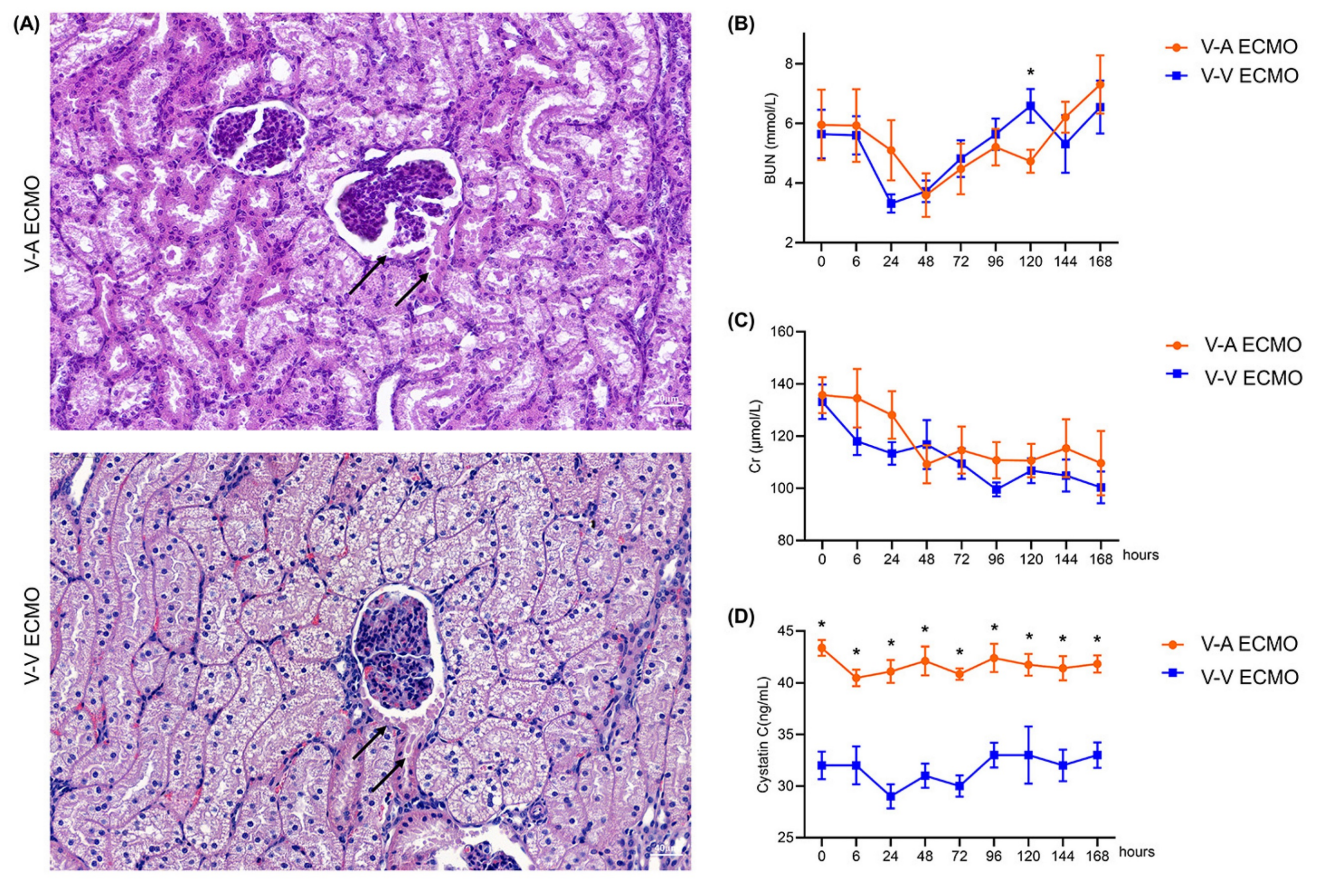
** The structure and function of the glomerulus in the ECMO model.** (A) The structure of the glomerulus in both groups. The black arrows point to the protein that has filtered into the renal capsule. Scale bar: 40 μm. The concentration of blood urea nitrogen (B) and creatinine (C) was tested throughout the procedure. After the experiment, the concentration of plasma cystatin C (D) was determined by enzyme-linked immunosorbent assay. (n = 5, each group). V-A ECMO: veno-arterial extracorporeal membrane oxygenation; V-V ECMO: veno-venous extracorporeal membrane oxygenation; BUN: blood urine nitrogen; Cr: creatinine. * *p* < 0.05; *p* > 0.05 was not shown.

**Figure 5:**
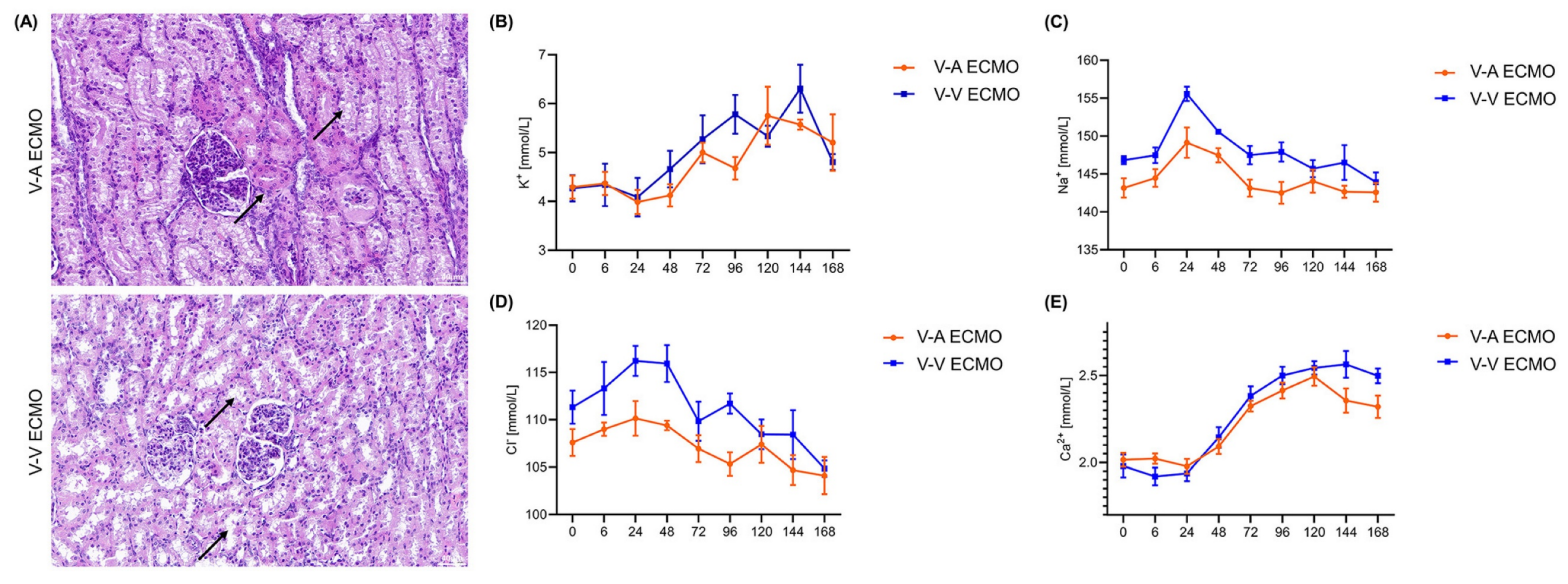
** The structure and function of the renal tubules in the ECMO model.** (A) The structure of the renal tubules in both groups. The black arrows in the V-A ECMO group point to the edema of the renal tubules and vacuole-like denaturation. The black arrows in the V-V ECMO group point to the protein casts. Scale bar: 40μm. The concentration of plasma K+ (B), Na+ (C), Cl- (D), and Ca2+ (E) was tested throughout the experiment. V-A ECMO: veno-arterial extracorporeal membrane oxygenation; V-V ECMO: veno-venous extracorporeal membrane oxygenation. *p* > 0.05 was not shown.

Protein-like substances were observed in renal sacs in both groups **(Figure 4A)**. The concentration of BUN and Cr were similar in both groups during the whole procedure **(Figure 4B-C; Table S2)**. However, the concentration of CyC was higher in the diminished pulsatile flow group since the initiation of ECMO **(Figure 4D; Table S2)**. The proximal tubules of the diminished pulsatile flow group showed edema and vacuole-like degeneration, while those of the normal group showed protein tubular type** (Figure 5A)**. However, the concentration of serum K^+^, Na^+^, Cl^-^, and Ca^2+^ were in the normal range and the trend was similar in both groups **(Figure 5B-E; Table S3)**.

### Early kidney injury was observed in both the two groups

**Figure 6:**
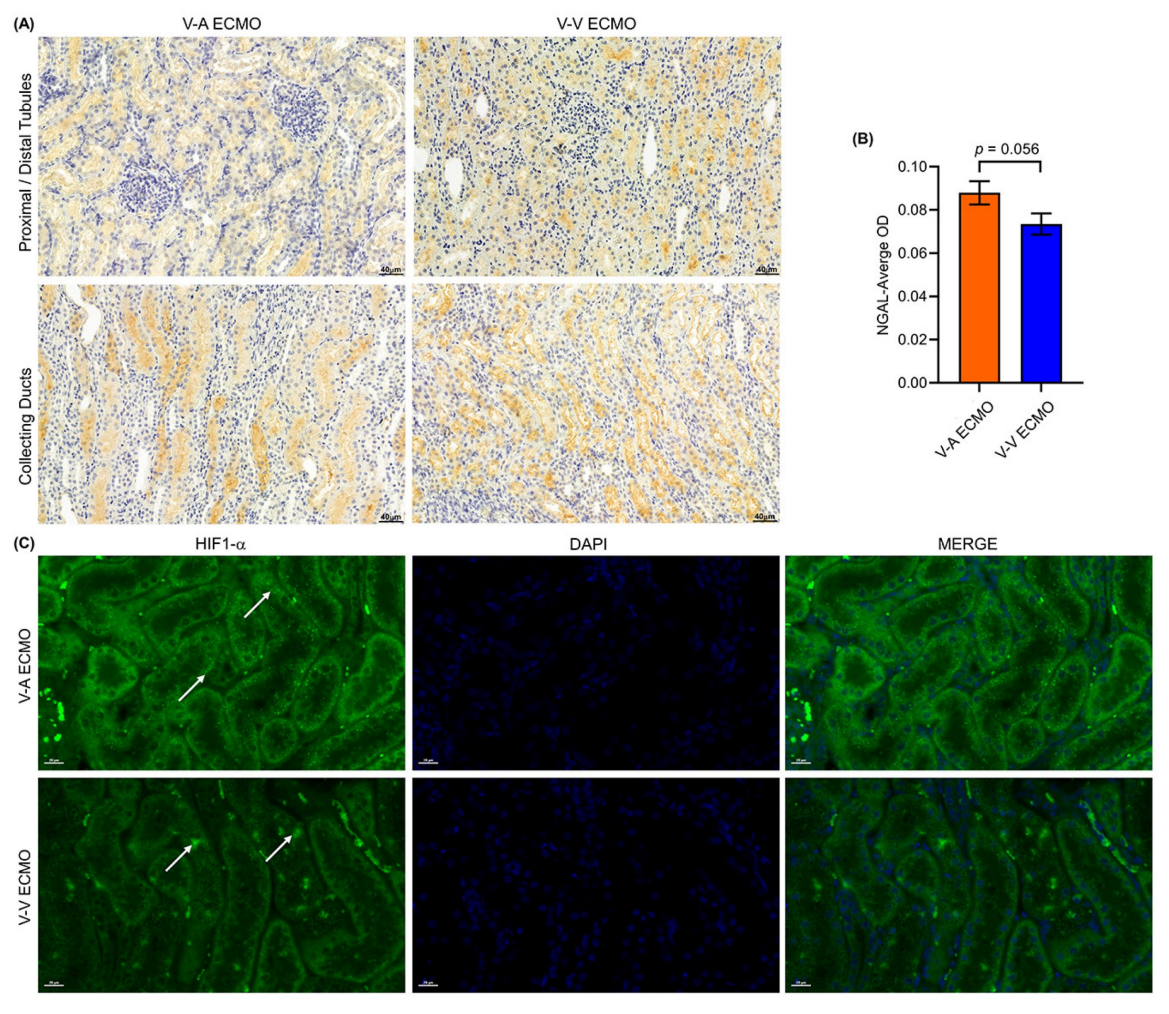
** Kidney injury and hypoxia in ECMO sheep.** (A) The expression of NGAL was tested in both groups. Three fields were randomly selected for each kidney specimen (n = 5, each group) and the average optical density (OD) was calculated using the Image J software. Scale bar: 40μm. The semi-quantitative expression of NGAL (B) was similar in the two groups (*p* = 0.056). The HIF-1α was detected using immunofluorescence (C). The white arrows show the HIF-1α protein in renal tubules. Scale bar: 20μm. V-A ECMO: veno-arterial extracorporeal membrane oxygenation; V-V ECMO: veno-venous extracorporeal membrane oxygenation.; NGAL: neutrophil gelatinase-associated lipocalin; HIF-1α: hypoxia inducible factor-1α.

To further investigate the kidney injury, we used immune-histochemical staining and immune-fluorescence staining to evaluate the tubule injury and hypoxia via NGAL and HIF1-α, respectively. There was a large amount of NGAL protein in the renal tubules and collecting tubules of the two groups** (Figure 6A)**. Semi-quantitative results show no significant difference in NGAL expression between the two groups** (Figure 6B)**. HIF1-α was expressed in renal tubules in both groups, indicating that the kidneys in both groups experienced hypoxia **(Figure 6C)**. A small focal renal infarction occurred in the kidney of one sheep in both groups.

## Discussion

The purpose of our study was to evaluate the specific impact of blood flow pulsatility under V-A ECMO condition on renal physiology so we established the V-A ECMO sheep model in comparison to V-V ECMO to control the single variable which was cannulation strategy. In both V-A ECMO and V-V ECMO groups, vital signs, anticoagulant indicators, and renal function biomarkers were all in the normal range during the whole experiment. However, diminished pulsatile flow influenced the expression of proteins in renal endothelial cells and the secretion of renin. Besides, the histopathologic findings demonstrated that early-stage kidney injuries, such as tubule injury protein accumulation and mild tubule hypoxia, have already occurred in both groups. Strengths of our study include being the first to study the impact of diminished pulsatile flow induced by V-A ECMO on renal endothelial cells and renin secretion; using health-conscious sheep to study the impact of V-A ECMO with the comparison of V-V ECMO; providing kidney biopsy specimens evidence to delineate AKI characteristics at the 7th day of ECMO management.

Pulsatile blood flow decreased the vascular tone significantly by regulating the endothelium-derived nitric oxide release^20^. While non-pulsatile blood flow increases systemic vascular resistance and oxygen consumption. Endothelial cells are recognized to play an important role in both modulating the vascular tone and coordinating tissue perfusion. Our results confirmed that under the condition of diminished pulsatile flow, the CD31 protein which is the marker of endothelial cells was down-regulated. Figuring out the underlying mechanism may protect the integrity and cellular function of the endothelial cells. A previous study found that the reduction in pulse pressure stimulates renin release, in which plasma renin activity was significantly elevated^21^. In our study, we failed to measure renin levels in plasma, but we found that the ratio of renin-positive glomerulus was lower in the diminished pulsatile flow group. The low ratio of renin-positive glomerulus is probably due to the release of renin from the paratropical apparatus into the blood. Although there was no difference in mean arterial pressure in the two groups, the diminished pulsatile flow had an impact on the regulation of the renin-angiotensin system. In 1979, James et al. figured out there was no difference in total renal cortical flow or in flow distribution using pulsatile compared with non-pulsatile perfusion when mean perfusion pressure was held around 50 - 80 mmHg^22^. In our study, there was no significant difference in renal function between the two groups after 7 days of ECMO support. These results indicated that pulsatility is one of the terms of blood perfusion, further study is still needed to depict the exact mechanism between pulsatility and renal function.

The impact of ECMO management on the kidney is multiple. Although we maintained MAP fluctuations at baseline during ECMO management, there was still partial HIF-1α expression within the renal tubules, which led us to recognize that perfusion pressure was not the only indicator of guaranteed oxygen delivery. In addition to the differences in blood pulsatility found in our study, different flow rates between the two groups may also affect renal pathophysiological processes. Continuous infusion of heparin during maintenance of ECMO can also affect the kidney. Whether the above-mentioned processes can induce HIF-1α expression by increasing oxygen consumption needs further investigation.

The serum concentration of Cr and urine output (UO) are the criteria for AKI detection^23^. However, both of them are markers for renal function, not kidney injury. Scr is a delayed and insensitive biomarker for it does not indicate the severity of kidney injury. Only using the Scr for identification of AKI is simplistic. It may erroneously imply specific pathophysiology, and location of the injury, potentially leading to therapeutic imprecision. As a measure of renal function, CyC was superior to SCr^24^. Our results also demonstrated that there was tubular injury under the normal range of SCr. Rajit L, et al. figured out that composites of functional damage biomarker (CyC) and tubular damage biomarker (NGAL) are superior to SCr for predicting AKI^25^. To enhance diagnostic precision, it is necessary to use novel biomarkers indicative of different AKI pathophysiological conditions in combination with functional biomarkers^26^-^28^.

The ultimate goal of the research is to better treat diseases and improve the prognosis of patients. We studied the healthy sheep kidney and found a correlation between the different blood pulsatility and the changes in renal function under different ECMO modes. Under the condition of V-A ECMO, the diminished pulsatile flow will affect the kidney, which suggests that maintaining normal pulsatile flow is positive for the kidney. The results led us to rethink the importance of blood flow pulsation for end-organ perfusion. It has not yet been fully elucidated that the specific physiological function of pulsatile blood flow so far. Our study is only the first step in investigating the specific physiological functions of blood flow pulsation. In future studies, we can establish disease models (such as the cardiogenic shock model) to evaluate the effect of diminished pulsatile flow on the injured kidney during V-A ECMO, the results obtained at that time will better reflect the clinical reality.

Although the useful findings, we encountered some limitations in this study. First, we did not use energy-related formulas to evaluate the pulsatility of blood flow^29^. The duration of ECMO management is 7 days and there was complexity in using energy-related formulas, so we used the common vital signs (PP, PI) instead. Second, the urine output was not calculated and the urine sample was not collected during the experiment. We failed to evaluate the content of urine, making the assessment of renal function and kidney injury incomplete. Besides, only male sheep were selected as the subjects of study. Because compared to male animals, female animals experience significant monthly variations in estrogen and progesterone levels, while the testosterone levels in male animals are relatively stable. Building on the results of this experiment, we will continue to monitor whether there are differences in similar studies between male and female animals in the future. Moreover, the early-stage kidney injury was confirmed via the biopsy, but further investigation is needed to figure out whether the injury is reversible or not. Additionally, although we found early kidney injury under the condition of diminished pulsatile flow through an ECMO model, further mechanism studies are also still needed to clarify the exact relationship between them.

## Conclusion

During the management of V-A ECMO or V-V ECMO, although the serum BUN and Cr were normal, there was exactly an early-stage injury of the glomerulus and tubules in both groups. Diminished pulsatile flow under the V-A ECMO condition can increased the concentration of cystatin C and increased the expression of CD31 compared with normal pulsatile flow supported by V-V ECMO. Future study is needed to optimize kidney protection during ECMO support. For instance, what degree of pulsatility should be maintained without potentially impaired endothelial function?

## Supplementary Material

Supplementary tables.

## Figures and Tables

**Table 1:** Characteristics of sheep in the two groups.

Variables	V-A ECMO (n=5)	V-V ECMO (n=5)	P-value
**Baseline**			
Weight, kg	56.00 ± 1.581	59.60 ± 2.51	0.027
HR, bpm	114.00 ± 28.59	142.00 ± 12.39	0.079
SBP, mmHg	114.6 ± 16.45	99.20 ± 6.91	0.122
DBP, mmHg	90.20 ± 15.28	82.00 ± 9.34	0.384
MAP, mmHg	93.00 ± 9.30	88.40 ± 9.50	0.461
Hb, g/L	93.60 ± 17.73	126.20 ± 24.69	0.043
BUN, mmol/L	6.27 ± 2.06	5.39 ± 1.50	0.324
Cr, μmol/L	158.57 ± 35.00	134.94 ± 14.03	0.199
**ECMO 6 hours**			
Pump Speed, rpm	3389.0 (3248.0, 3498.0)	33498.0 (3496.5, 3498.0)	0.075
Flow, L/min	2.00 (2.00, 2.45)	2.00 (1.70, 2.05)	0.079
Pre-pump Pressure, mmHg	-45.00 ± 5.94	-66.00 ± 3.67	< 0.001
Post-pump pressure, mmHg	178.20 ± 14.65	167.40 ± 11.87	0.236
ACT, s	252.00 ± 44.51	248.40 ± 53.76	0.911
**Weaning from ECMO**			
Pump Speed, rpm	3248.0 (3198.0, 3500.0)	3498.0 (3497.0, 3498.5)	0.043
Flow, L/min	2.08 ± 0.28	1.84 ± 0.15	0.128
Pre-pump Pressure, mmHg	-44.60 ± 20.45	-67.40 ± 9.48	0.054
Post-pump pressure, mmHg	166.0 (144.5, 195.0)	163.0 (152.0, 167.5)	0.509
ACT, s	237.40 ± 27.90	258.20 ± 39.58	0.368
HR, bpm	119.40 ± 26.04	115.00 ± 48.06	0.863
SBP, mmHg	116.60 ± 19.28	112.80 ± 11.39	0.743
DBP, mmHg	91.60 ± 18.68	85.80 ± 4.66	0.565
MAP, mmHg	107.00 ± 22.79	105.40 ± 8.76	0.889
Hb, g/L	97.60 ± 14.90	91.20 ± 10.04	0.451
Free Hb, g/L	0.1 (0.05, 0.25)	0.1 (0.1, 0.2)	0.363

HR: heart rate; MAP: mean arterial pressure; Hb: hemoglobin; BUN: blood urine nitrogen; Cr: creatinine; ECMO: extracorporeal membrane oxygenation; ACT: activated clotting time.

## Abbreviations

ACT: activated clotting time
BUN: blood urea nitrogen
CD31: anti-platelet endothelial cell adhesion molecule-1
Cr: creatinine
CyC: cystatin C
DLC: double-lumen cannula
ECMO: extracorporeal membrane oxygenation
Hb: hemoglobin
HIF-1α: hypoxia-inducible factor-1α
MAP: mean arterial pressure
NGAL: neutrophil gelatinase-associated lipocalin
PaO2: partial pressure of arterial oxygen
PI: pulsatility index
PP: pulse pressure
SD: standard deviations
V-A ECMO: veno-arterial extracorporeal membrane oxygenation
V-V ECMO: veno-venous extracorporeal membrane oxygenation
