# Supplementary material for: Kidney Injury under Diminished Pulsatile Flow Induced by V-A ECMO in Sheep
Source: Int J Med Sci. 2025 Jan 27;22(4):971–81. doi: 10.7150/ijms.103349 (PMC11843139; doi:10.7150/ijms.103349)
Supplement: Supplementary file 1 — Supplementary tables. [file ijmsv22p0971s1.pdf]

Supplementary Table 1: Statistical comparison results of basic vital signs.

Time point	Mean of V-A EMCO	Mean of V-V EMCO	Difference	Standard Deviation of Difference	P value
			**Heart Rate**		
0	114	142	-28	12.630	0.0574
12	94	103	-9	19.030	0.6488
24	80	102	-22	16.830	0.2276
36	74	96	-22	13.150	0.1329
48	68	87	-19	8.877	0.0647
60	75	82	-7	8.683	0.4435
72	81	98	-17	13.000	0.2273
84	94	94	0	12.660	1.0000
96	87	103	-16	11.220	0.1916
108	134	103	31	23.770	0.2284
120	86	128	-42	20.260	0.0719
132	118	128	-10	18.730	0.6079
144	105	119	-14	19.420	0.4914
156	147	119	28	26.340	0.3188
168	150	119	31	30.420	0.3380
			**Mean Arterial Pressure**		
0	93	88	5	5.060	0.3520
12	96	105	-9	3.225	**0.0235**
24	91	99	-8	8.683	0.3838
36	97	98	-1	6.826	0.8872
48	98	102	-4	8.602	0.6543
60	102	104	-2	4.754	0.6850
72	98	106	-8	7.810	0.3357

84	103	104	-1	9.818	0.9214
96	96	100	-4	8.062	0.6331
108	97	104	-7	6.000	0.2769
120	98	97	1	7.642	0.8991
132	110	101	9	8.786	0.3357
144	105	100	5	7.655	0.5320
156	104	101	3	8.222	0.7247
168	102	96	6	8.809	0.5150
**Pulse Pressure**					
0	20.4	34.4	-14	5.798	**0.0422**
12	20.4	36	-15.6	7.782	0.0799
24	25.2	29	-3.8	7.658	0.6331
36	24.2	27.2	-3	6.556	0.6594
48	19.6	33.6	-14	4.787	**0.0192**
60	22.2	29.4	-7.2	6.826	0.3224
72	22.6	30.6	-8	6.551	0.2568
84	16.2	30.6	-14.4	4.472	**0.0122**
96	16.4	29	-12.6	4.366	**0.0203**
108	21.4	27	-5.6	4.966	0.2921
120	18.8	26	-7.2	7.736	0.3792
132	23.4	27.8	-4.4	4.868	0.3925
144	19.2	24	-4.8	4.532	0.3205
156	22	26.6	-4.6	3.156	0.1831
168	19	30.8	-11.8	5.526	0.0653
**Puisatility Index**					
12	0.2135	0.3377	-0.1242	0.071	0.1204

24	0.2719	0.2957	-0.02387	0.076	0.7606
36	0.2526	0.277	-0.02445	0.071	0.7406
48	0.2012	0.329	-0.1277	0.042	**0.0155**
60	0.2148	0.2825	-0.06763	0.063	0.3158
72	0.2284	0.2887	-0.06021	0.056	0.3102
84	0.1634	0.2965	-0.1331	0.048	**0.0234**
96	0.1678	0.2925	-0.1248	0.041	**0.0158**
108	0.2147	0.2594	-0.04476	0.041	0.3104
120	0.1904	0.2613	-0.07094	0.069	0.3322
132	0.2149	0.2744	-0.05947	0.045	0.2266
144	0.1815	0.2401	-0.05851	0.040	0.1862
156	0.2214	0.2638	-0.04242	0.042	0.3382
168	0.1857	0.3209	-0.1353	0.054	**0.0379**

Supplementary Table 2: Statistical comparison results of kidney function indicators.

Time point	Mean of V-A EMCO	Mean of V-V EMCO	Difference	Standard Deviation of Difference	P value
			**Blood urea nitrogen**		
0	5.9460	5.6360	0.3100	1.4350	0.8344
6	5.9200	5.5940	0.3260	1.3800	0.8191
24	5.0920	3.3100	1.7820	1.0580	0.1305
48	3.5920	3.7200	-0.1280	0.8186	0.8796
72	4.4720	4.8140	-0.3420	1.0500	0.7529
96	5.2000	5.6320	-0.4320	0.8094	0.6080
120	4.7300	6.5800	-1.8500	0.6884	**0.0276**
144	6.2000	5.3020	0.8980	1.1000	0.4377
168	7.3020	6.5400	0.7620	1.3220	0.5802
			**Creatinine**		
0	135.7	133.2	2.48	9.583	0.80233
6	134.5	118	16.45	12.4	0.221201
24	128.1	113.4	14.73	10.16	0.185241
48	109.2	116.8	-7.57	11.88	0.541884
72	114.6	109.4	5.2	10.75	0.641401
96	110.8	99.54	11.21	7.458	0.171213
120	110.7	106.8	3.85	7.994	0.642962
144	115.4	104.9	10.46	12.66	0.432758
168	109.7	100.3	9.32	13.77	0.517604
			**Plasma cystatin C**		
0	43.37	32.53	10.84	1.544	**0.00011**
6	40.49	32.62	7.87	2.017	**0.004528**
24	41.11	29.2	11.91	1.604	**0.000074**

48	42.12	31.79	10.33	1.821	**0.000469**
72	40.86	30.25	10.61	1.179	**0.000018**
96	42.41	33.69	8.72	1.821	**0.001375**
120	41.75	33.67	8.08	2.978	**0.026538**
144	41.42	32.58	8.84	1.937	**0.001838**
168	41.84	33.26	8.58	1.498	**0.000441**

Supplementary Table 3: Statistical comparison results of electrolyte value.

Time point	Mean of V-A EMCO	Mean of V-V EMCO	Difference	Standard Deviation of Difference	P value
			**K+**		
0	4.2920	4.2680	0.0240	0.3579	0.9482
6	4.3680	4.3400	0.0280	0.4483	0.9522
24	3.9900	4.0880	-0.0980	0.4658	0.8386
48	4.1240	4.6620	-0.5380	0.4379	0.2542
72	5.0020	5.2700	-0.2680	0.5323	0.6282
96	4.6780	5.7820	-1.1040	0.4581	0.0425
120	5.7520	5.3360	0.4160	0.6314	0.5285
144	5.5720	6.3040	-0.7320	0.5022	0.1831
168	5.2060	4.8060	0.4000	0.6002	0.5239
			**Na^+**		
0	143.1	146.8	-3.668	1.388	0.029583
6	144.5	147.5	-3	1.55	0.088975
24	149.1	155.6	-6.452	2.228	0.020018
48	147.5	150.6	-3.098	0.9783	0.013262
72	143.1	147.5	-4.344	1.658	0.030636
96	142.5	147.9	-5.4	1.915	0.022503
120	144.1	145.7	-1.642	1.906	0.413939
144	142.7	146.5	-3.864	2.435	0.151179
168	142.6	143.9	-1.362	1.786	0.467619
			**Cl^-**		
0	107.6	111.3	-3.726	2.255	0.137111
6	109	113.3	-4.322	2.894	0.173658
24	110.1	116.2	-6.082	2.441	0.037423

48	109.4	115.9	-6.561	2.255	0.022696
72	106.9	109.8	-2.897	2.421	0.270364
96	105.3	111.7	-6.386	1.641	0.004597
120	107.4	108.5	-1.064	2.511	0.682959
144	104.7	108.4	-3.744	3.028	0.251298
168	104.1	104.8	-0.74	2.137	0.738036
**Ca^{+}**					
0	2.016	1.98	0.036	0.07301	0.637048
6	2.022	1.92	0.102	0.05886	0.121319
24	1.978	1.938	0.04	0.06155	0.533962
48	2.092	2.144	-0.052	0.07348	0.499268
72	2.324	2.382	-0.058	0.06387	0.390398
96	2.414	2.5	-0.086	0.06867	0.245827
120	2.494	2.544	-0.05	0.06574	0.468747
144	2.356	2.564	-0.208	0.1042	0.080931
168	2.32	2.498	-0.178	0.07703	0.049634
